# Global gender disparities in editorial leadership of radiology journals: a cross-sectional analysis of bibliometric and economic associations

**DOI:** 10.1186/s13244-025-02128-w

**Published:** 2025-10-30

**Authors:** Paola Martinez-Greiser, Ernesto Roldan-Valadez, Sergey K. Ternovoy, Filiberto Toledano-Toledano

**Affiliations:** 1 Directorate of Research, Hospital General de Mexico ‘Dr. Eduardo Liceaga’, Mexico City, Mexico; 2 Division of Research, Instituto Nacional de Rehabilitación “Luis Guillermo Ibarra Ibarra”, Mexico City, Mexico; 3 Department of Radiology, I.M. Sechenov First Moscow State Medical University (Sechenov University), Moscow, Russia; 4 A.L. Myasnikov Research Institute of Clinical Cardiology of National Medical Research Center of Cardiology of the Ministry of Health of Russia, Moscow, Russia; 5 Unidad de Investigación en Medicina Basada en Evidencias, Hospital Infantil de México Federico Gómez Instituto Nacional de Salud, Mexico City, Mexico; 6 Unidad de Investigación Multidisciplinaria en Salud, Instituto Nacional de Rehabilitación “Luis Guillermo Ibarra Ibarra”, Mexico City, Mexico; 7 Dirección de Investigación y Diseminación del Conocimiento, Instituto Nacional de Ciencias e Innovación para la Formación de Comunidad Científica, INDEHUS, Mexico City, Mexico

**Keywords:** Gender equity, Editorial board, Radiology journals, Bibliometric analysis, Diversity in leadership

## Abstract

**Objectives:**

To evaluate gender representation among editors-in-chief and deputy editors of radiology journals indexed in the 2024 *Journal Citation Reports* (JCR) and to analyze associations with bibliometric indicators and global economic classification.

**Materials and methods:**

A cross-sectional study was performed using publicly available data from radiology-related journals listed in the 2024 JCR (released June 2025). Journals were included if the editorial board composition was accessible online. Gender was identified through institutional profiles and standardized databases. Descriptive statistics summarized gender distribution. Associations between gender, editorial role, bibliometric performance, and World Bank income classification were tested using chi-square, Mann–Whitney U, Spearman’s correlation, and nominal logistic regression.

**Results:**

Of 204 eligible journals, 135 met the inclusion criteria, comprising 387 editorial members. Women represented 20.2% of all editors, 21.4% of deputy editors, and 18.4% of editors-in-chief. Female representation was highest in Q1 journals (26.0%) and lowest in Q2 (15.1%). A significant association was observed between Eigenfactor Score and female representation (*p* = 0.0494), whereas no association was found with journal impact factor or income classification. Geographic disparities were evident, with some countries achieving parity while others had no female representation.

**Conclusions:**

Gender inequities remain pronounced in radiology editorial leadership, particularly at the editor-in-chief level. Higher Eigenfactor Scores may modestly correlate with improved inclusion. Transparent policies and targeted interventions are required to address structural inequities and advance diversity in academic publishing.

**Critical relevance statement:**

Gender disparities exist in radiology editorial leadership, and the Eigenfactor Score was found to be associated with female representation. By providing a comprehensive overview, the findings underscore the structural barriers that limit diversity and the importance of transparent, equity-focused editorial policies.

**Key Points:**

- Gender disparities persist in radiology editorial boards, with women underrepresented at both deputy editor and editor-in-chief levels.
- Eigenfactor Score, but not impact factor or national income classification, was significantly associated with increased female representation.
- Gender disparities persist across editorial leadership roles in radiology, underscoring the need for transparent policies and structural reforms to promote greater equity.

**Graphical Abstract:**

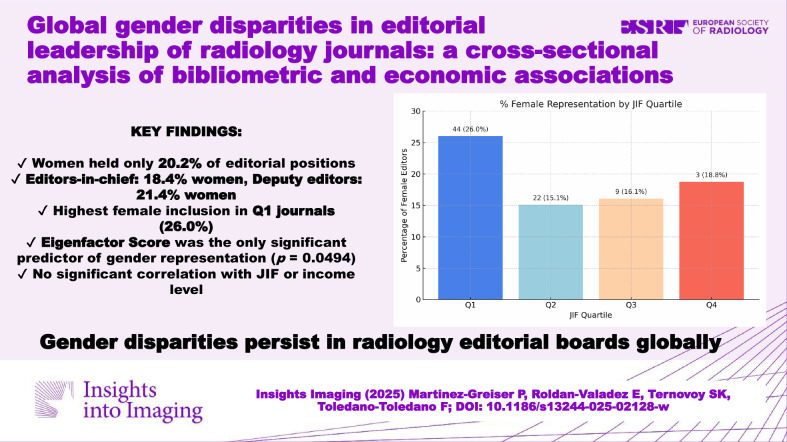

## Introduction

Gender diversity in academic leadership enhances innovation, promotes equity, and strengthens institutional decision-making [1]. In radiology and medical imaging, however, women remain markedly underrepresented in senior editorial roles, despite steady increases in the number of female medical graduates and specialists [1–4]. Editorial boards are central to shaping research agendas, setting peer review standards, and guiding scholarly discourse. Limited gender diversity in these positions raises concerns about systemic bias and inclusivity in scientific publishing [1, 5, 6].

Several studies highlight this imbalance. Alkhawtani et al [1] found that women accounted for only 8.8% of editors-in-chief and 21.5% of board members across 57 radiology journals with impact factors above 2.0 [1]. These results suggest persistent underrepresentation, with little correlation between gender diversity and journal impact, indicating that prestige alone does not guarantee equity. Similarly, Campos et al reported that women occupied only 17% of editorial roles in Latin American surgical and anesthesia journals, underscoring structural barriers such as the “glass ceiling” [2, 4].

Evidence suggests that gender-diverse editorial boards are associated with higher-quality publications, broader perspectives, and more effective decision-making [2]. Inclusive teams foster innovation, increase citations, and enhance organizational efficiency [5]. Thus, gender equity is not only a social priority but also integral to academic excellence.

Despite growing recognition, data on the gender composition of editorial boards across the full spectrum of radiology journals indexed in the Journal Citation Reports (JCR) remain limited. Prior studies analyzed fewer than half of the journals in the “Radiology, Nuclear Medicine & Medical Imaging” category, restricting insights into how gender representation relates to bibliometric performance and geographic or economic diversity [7, 8]. Moreover, the relationship between editorial board composition and World Bank country income levels remains poorly understood.

This study aimed to provide a comprehensive assessment of gender representation on editorial boards of JCR-indexed radiology journals as of 2024. Specifically, we analyzed the distribution of editors-in-chief and deputy editors and evaluated associations with journal impact factor, Eigenfactor score, geographic region, and national income classification. Our findings seek to inform editorial policy, advance inclusivity, and support systemic reforms to promote gender equity in academic radiology.

## Materials and methods

### Study design and eligibility criteria

This retrospective cross-sectional study assessed gender diversity within the editorial boards of *Radiology, Nuclear Medicine & Medical Imaging* journals indexed in the Science Citation Index Expanded (SCI-E) of the 2024 *Journal Citation Reports* (JCR) [9]. Reporting adhered to the STROBE (Strengthening the Reporting of Observational Studies in Epidemiology) guidelines [10].

Journals were eligible if they:

- Were listed under the “Radiology, Nuclear Medicine & Medical Imaging” category in JCR 2024;
- Had an active publication status during data collection;
- Provided publicly accessible editorial board information on official websites.

Exclusion criteria included incomplete or outdated editorial information, journals primarily focused on other disciplines (e.g., radiation oncology or medical physics), and editorial members with undetermined gender after exhaustive verification. Honorary, emeritus, or administrative positions were excluded. For listings with initials only, gender was determined using institutional profiles, author pages, or academic publications; unresolved cases were excluded.

### Ethical considerations

As only publicly available data were analyzed and no human participants were involved, formal ethical approval was not required.

### Data collection

A comprehensive JCR 2024 search identified 204 journals, of which 135 met the inclusion criteria. Editorial board members were classified as editors-in-chief or deputy editors based on official website listings. Data were collected between March and April 2025.

Bibliometric indicators were retrieved from JCR 2023 and included: Impact Factor (IF), Immediacy Index, Eigenfactor Score, Article Influence Score, Cited Half-Life, Total Citations, and Total Articles. Definitions followed standard bibliometric references [11].

Gender classification was conducted according to the SAGER (Sex and Gender Equity in Research) guidelines, which conceptualize gender as a social construct. Gender was determined using names, professional photographs, institutional biographies, and public databases; in ambiguous cases, automated tools such as Genderize.io were used [4, 12]. Unresolved cases were excluded. Due to limitations of available data and classification tools, gender was analyzed within a binary male/female framework, acknowledging that this methodological constraint does not capture the full gender spectrum.

### Geographic and economic classification

Geographic origin was defined by the country of primary institutional affiliation. Countries were categorized using the 2024 World Bank income classification (low-, lower-middle-, upper-middle-, and high-income economies), based on gross national income per capita [13]. Analyses were stratified at both journal and editor levels.

### Statistical analysis

Descriptive statistics were reported as counts and percentages to characterize gender representation across editorial roles, countries, and journal quartiles.

- The Mann–Whitney U test compared Journal Impact Factor (JIF) values between male and female editors-in-chief.
- Chi-square (χ²) tests assessed differences in gender distribution across roles, JIF quartiles, income groups, and countries.
- One-way analysis of variance compared bibliometric indicators across JIF quartiles.
- Spearman’s rank correlation examined associations between JIF quartile and female representation.

A binary logistic regression estimated the likelihood of female editorial representation based on Eigenfactor Score, JIF quartile, and editorial role. Country income classification was excluded due to incomplete data. Model fit was assessed with the likelihood ratio test, and pseudo R² values were reported. Stratified logistic regression further examined the probability of female representation as a function of Eigenfactor Score, stratified by editorial role. Predicted probabilities were visualized using logistic regression curves.

Statistical significance was set at *p* < 0.05. Analyses were performed using JMP® (JMP Statistical Software) Pro version 17.1.0 (SAS Institute Inc., Cary, NC, USA) and R version 4.3.2.

### Software

Microsoft Excel (v16.33) was used for initial data organization and cleaning. Statistical analyses were performed in JMP® Pro and R. Logistic regression models and visualizations were generated in Python using matplotlib, seaborn, scikit-learn, and statsmodels. Figures were refined for publication clarity and resolution.

## Results

### Journal selection and cohort description

Of 204 journals listed under *Radiology, Nuclear Medicine, and Medical Imaging* in the 2024 *Journal Citation Reports* (JCR), 135 (66.2%) met eligibility criteria. Sixty-nine journals (33.8%) were excluded due to incomplete or inaccessible editorial board data. The final cohort included 387 editorial board members, stratified by role, gender, country, and bibliometric profile.

### Bibliometric trends by JIF quartile

Bibliometric indicators varied significantly across journal impact factor (JIF) quartiles (Table 1).

- Q1 journals had the strongest metrics (median JIF = 3.5, Article Influence Score = 1.22, median articles = 301, citations = 19,673).
- Q2 journals showed intermediate performance (median JIF = 2.4, Article Influence Score = 0.73).
- Q3 journals displayed lower metrics (median JIF = 1.5) but longer cited half-life (6.90), suggesting citation longevity.
- Q4 journals recorded the lowest JIF (0.9) and Article Influence Score (0.28), yet the longest cited half-life (8.45), reflecting sustained niche relevance.

**Table 1:** Bibliometric indicators by journal impact factor (JIF) quartile

JIF quartile	Median JIF	Median immediacy index	Median Eigenfactor	Median article influence score	Median cited half-life	Median total articles	Median total citations	Median JIF without self-cites	Median normalized Eigenfactor
Q1	3.5	1.0	0.02	1.22	7.4	301.0	19,673.0	3.4	3.97
Q2	2.4	0.5	0.01	0.73	4.9	181.0	5561.0	2.2	1.38
Q3	1.5	0.3	0.0	0.44	6.9	77.5	1687.0	1.4	0.3
Q4	0.9	0.25	0.0	0.28	8.45	62.0	708.0	0.9	0.13

Median values and interquartile ranges for JIF, Article influence score, Eigenfactor score, total articles, total citations, and cited half-life across journals in JIF quartiles Q1 to Q4

### Gender representation across editorial boards

Among 387 editors, women comprised 20.16% (*n* = 78) compared to 79.84% men (*n* = 309), a statistically significant disparity (χ² = 273.385, *p* < 0.0001).

- Editors-in-Chief (EIC): 30 women (18.40%) vs. 133 men (χ² = 127.656, *p* < 0.0001).
- Deputy Editors: 48 women (21.43%) vs. 176 men (χ² = 144.009, *p* < 0.0001).

These findings highlight the persistent underrepresentation of women in journal leadership.

### Gender distribution by JIF quartile

**Fig. 1:**
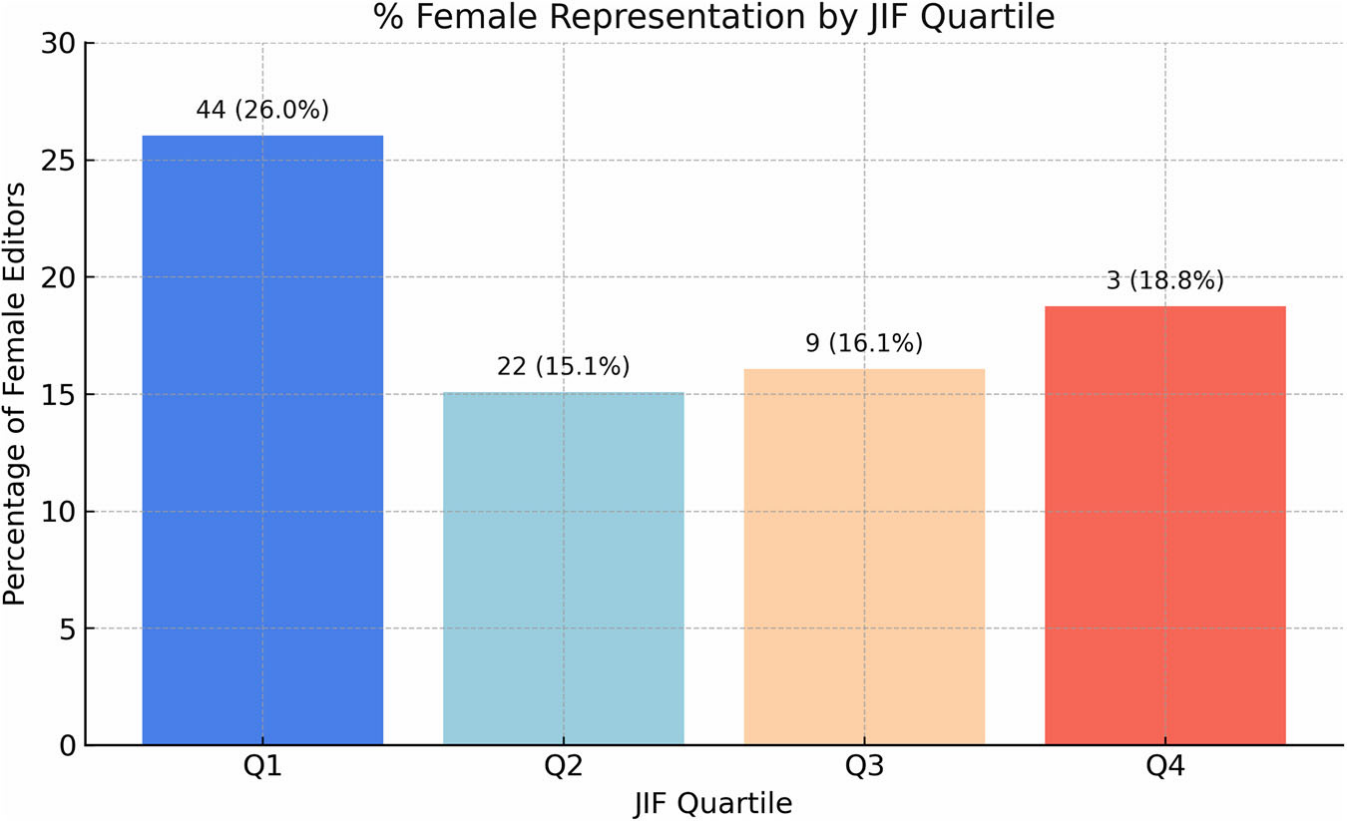
Female representation by journal impact factor (JIF) quartile. Bar chart showing the percentage and count of female editors (combined editors-in-chief and deputy editors) across JIF quartiles (Q1 to Q4). Female representation was highest in Q1 journals (26.0%) and lowest in Q2 (15.1%), with modest variability across quartiles

Female representation across quartiles is shown in Fig. 1 and Table 2:

- Q1: 26.0% (*n* = 44/169)
- Q2: 15.1% (*n* = 22/146)
- Q3: 16.1% (*n* = 9/56)
- Q4: 18.8% (*n* = 3/16)

**Table 2:** Female editor representation by JIF quartile

JIF quartile	Female editors	Male editors	Total editors	% Female	Quartile rank
Q1	44.0	125.0	169.0	26.04	1.0
Q2	22.0	124.0	146.0	15.07	2.0
Q3	9.0	47.0	56.0	16.07	3.0
Q4	3.0	13.0	16.0	18.75	4.0

Number and percentage of female editors (combined editors-in-chief and deputy editors) by JIF quartile. Quartile-based distribution illustrates disparities in representation, with higher inclusion noted in Q1 journals

Statistical tests: Chi-square χ² = 6.58, *p* = 0.0866; Spearman correlation r = −0.20, *p* = 0.8000

The overall chi-square test was not significant (χ² = 6.58, *p* = 0.0866). Spearman’s correlation suggested a weak, non-significant inverse trend (r = −0.20, *p* = 0.8000), indicating higher-tier journals may be modestly more inclusive.

### Global gender distribution by country

Editorial members represented 42 countries across six continents (Table 3).

- The United States accounted for 35.9% (*n* = 139) of editors, with 23.0% women.
- High female representation was observed in Romania (2/2, full representation), Israel (66.7%), Denmark (50%), and Singapore (50%).
- Zero female editors were reported in Belgium, Brazil, Japan, Turkey, the United Arab Emirates, and South Africa.
- Moderate representation was found in Canada (30.8%), Italy (28.5%), and the UK (23.8%).

**Table 3:** Geographic distribution of editorial board members by country and gender

Country	Total % (*N*)	Female	Male
Australia	2.3% (9)	22.2% (2)	77.8% (7)
Austria	2.1% (8)	25.0% (2)	75.0% (6)
Belgium	1.0% (4)	0.0% (0)	100.0% (4)
Brazil	0.3% (1)	0.0% (0)	100.0% (1)
Canada	3.4% (13)	30.8% (4)	69.2% (9)
Chile	0.3% (1)	0.0% (0)	100.0% (1)
China	2.8% (11)	18.2% (2)	81.8% (9)
Denmark	0.5% (2)	50.0% (1)	50.0% (1)
France	6.7% (26)	15.4% (4)	84.6% (22)
Germany	10.1% (39)	12.8% (5)	87.2% (34)
Greece	0.5% (2)	0.0% (0)	100.0% (2)
Hong Kong	0.3% (1)	0.0% (0)	100.0% (1)
Hungary	0.3% (1)	0.0% (0)	100.0% (1)
India	0.5% (2)	0.0% (0)	100.0% (2)
Iran	0.5% (2)	0.0% (0)	100.0% (2)
Israel	0.8% (3)	66.7% (2)	33.3% (1)
Italy	4.6% (18)	28.5% (4)	75.0% (14)
Japan	5.9% (23)	0.0% (0)	100.0% (23)
Korea	2.3% (9)	44.4% (4)	55.6% (5)
New Zealand	0.3% (1)	0.0% (0)	100.0% (1)
Oman	0.3% (1)	0.0% (0)	100.0% (1)
Poland	0.3% (1)	0.0% (0)	100.0% (1)
Portugal	0.3% (1)	0.0% (0)	100.0% (1)
Romania	0.5% (2)	100.0% (2)	0.0% (0)
Scotland	0.3% (1)	0.0% (0)	100.0% (1)
Singapore	0.5% (2)	50.0% (1)	50.0% (1)
Slovenia	2.3% (9)	22.2% (2)	77.8% (7)
South Africa	0.3% (1)	0.0% (0)	100.0% (1)
Spain	0.8% (3)	33.3% (1)	66.7% (2)
Sweden	1.0% (4)	25.0% (1)	75.0% (3)
Switzerland	3.1% (12)	25.0% (3)	75.0% (9)
Taiwan	0.3% (1)	0.0% (0)	100.0% (1)
The Netherlands	2.6% (10)	10.0% (1)	90.0% (9)
Turkey	0.5% (2)	0.0% (0)	100.0% (2)
United Arab Emirates	0.3% (1)	0.0% (0)	100.0% (1)
United Kingdom	5.4% (21)	23.8% (5)	76.2% (16)
United States of America	35.9% (139)	23.0% (32)	77.0% (107)

Number and percentage of male and female editors across countries contributing to the editorial boards. Countries are ranked by total contribution. The percentage column reflects the share of each country’s representation within the total editor population analyzed

These variations suggest that sociocultural and institutional factors strongly influence editorial gender equity.

### Gender by editorial role and country

**Fig. 2:**
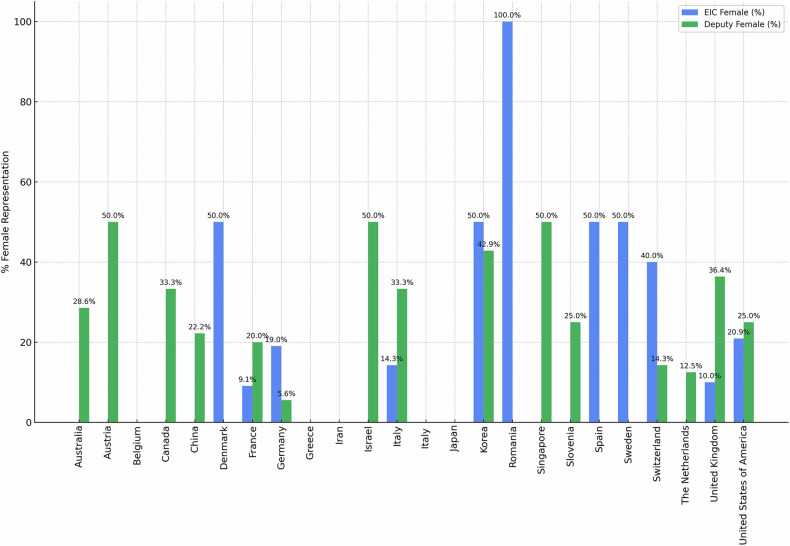
Female representation in editors-in-chief and deputy editor roles by country. Grouped bar chart depicting the percentage of female editors-in-chief (blue) and deputy editors (green) across countries contributing to radiology editorial boards. Substantial variability was observed, with countries such as Romania and Israel showing parity or dominance of female editors, while others, including Japan and Belgium, reported no female editorial leadership

Figure 2 and Table 4 illustrate gender stratification by role and geography.

- The United States contributed 41.1% (*n* = 67) of EICs, with 20.9% female representation.
- Countries with full parity at the EIC level included Romania (2/2, 100%), while Israel reported one female editor-in-chief (*n* = 1, full representation).
- Among deputies, Austria, Singapore, and Spain reported 50% female representation.
- Japan reported no women across 23 positions.

**Table 4:** Female representation by editorial role and country

Country	EIC total % (*N*)	EIC female	EIC male	Deputy total % (*N*)	Deputy female	Deputy male
Australia	1.2% (2)	0.0% (0)	100.0% (2)	3.1% (7)	28.6% (2)	71.4% (5)
Austria	2.5% (4)	0.0% (0)	100.0% (4)	1.8% (4)	50.0% (2)	50.0% (2)
Belgium	1.8% (3)	0.0% (0)	100.0% (3)	0.4% (1)	0.0% (0)	100.0% (1)
Canada	0.6% (1)	0.0% (0)	100.0% (1)	5.4% (12)	33.3% (4)	66.7% (8)
China	1.2% (2)	0.0% (0)	100.0% (2)	4.0% (9)	22.2% (2)	77.8% (7)
Denmark	1.2% (2)	50.0% (1)	50.0% (1)	—	—	—
France	6.7% (11)	9.1% (1)	90.9% (10)	6.7% (15)	20.0% (3)	80.0% (12)
Germany	12.9% (21)	19.0% (4)	81.0% (17)	8.0% (18)	5.6% (1)	94.4% (17)
Hong Kong	0.6% (1)	0.0% (0)	100.0% (1)	—	—	—
India	0.6% (1)	0.0% (0)	100.0% (1)	0.4% (1)	0.0% (0)	100.0% (1)
Iran	1.2% (2)	0.0% (0)	100.0% (2)	—	—	—
Israel	0.6% (1)	100.0% (1)	0.0% (0)	0.9% (2)	50.0% (1)	50.0% (1)
Italy	5.5% (9)	12.5% (1)	85.7% (8)	4.0% (9)	33.3% (3)	66.7% (6)
Japan	3.7% (6)	0.0% (0)	100.0% (6)	7.6% (17)	0.0% (0)	100.0% (17)
Korea	1.2% (2)	50.0% (1)	50.0% (1)	3.1% (7)	42.9% (3)	57.1% (4)
New Zealand	0.6% (1)	0.0% (0)	100.0% (1)	—	—	—
Poland	0.6% (1)	0.0% (0)	100.0% (1)	—	—	—
Romania	1.2% (2)	100.0% (2)	0.0% (0)	—	—	—
Slovenia	0.6% (1)	0.0% (0)	100.0% (1)	3.6% (8)	25.0% (2)	75.0% (6)
South Africa	0.6% (1)	0.0% (0)	100.0% (1)	—	—	—
Spain	1.2% (2)	50.0% (1)	50.0% (1)	0.4% (1)	0.0% (0)	100.0% (1)
Sweden	1.2% (2)	50.0% (1)	50.0% (1)	0.9% (2)	0.0% (0)	100.0% (2)
Switzerland	3.1% (5)	40.0% (2)	60.0% (3)	3.1% (7)	14.3% (1)	85.7% (6)
The Netherlands	1.2% (2)	0.0% (0)	100.0% (2)	3.6% (8)	12.5% (1)	87.5% (7)
Turkey	0.6% (1)	0.0% (0)	100.0% (1)	0.4% (1)	0.0% (0)	100.0% (1)
United Kingdom	6.1% (10)	10.0% (1)	90.0% (9)	4.9% (11)	36.4% (4)	63.6% (7)
United States of America	41.1% (67)	20.9% (14)	79.1% (53)	32.1% (72)	25.0% (18)	75.0% (54)
Brazil	—	—	—	0.4% (1)	0.0% (0)	100.0% (1)
Chile	—	—	—	0.4% (1)	0.0% (0)	100.0% (1)
Greece	—	—	—	0.9% (2)	0.0% (0)	100.0% (2)
Hungary	—	—	—	0.4% (1)	0.0% (0)	100.0% (1)
Oman	—	—	—	0.4% (1)	0.0% (0)	100.0% (1)
Portugal	—	—	—	0.4% (1)	0.0% (0)	100.0% (1)
Scotland	—	—	—	0.4% (1)	0.0% (0)	100.0% (1)
Singapore	—	—	—	0.9% (2)	50.0% (1)	50.0% (1)
Taiwan	—	—	—	0.4% (1)	0.0% (0)	100.0% (1)
United Arab Emirates	—	—	—	0.4% (1)	0.0% (0)	100.0% (1)

Distribution of female editors-in-chief and deputy editors across selected countries. Results highlight cross-national variability in female leadership, with full gender parity observed in some countries and a complete absence in others

While deputy roles showed progress in some regions, EIC positions remained largely male-dominated.

### Gender representation by World Bank income group

As summarized in Table 5:

- High-income countries (*n* = 313; 81.5%) reported 22.3% female deputies and 18.0% female EICs.
- Upper-middle-income countries (*n* = 59; 15.4%) reported only two female EICs (both from Romania) and three female deputies.
- Lower-middle-income countries (*n* = 12; 3.1%) showed no female editors.

**Table 5:** Gender representation by World Bank income classification and editorial role

Country	Income level	% (*N*)	Deputy female	Deputy male	EIC female	EIC male
Australia	High income	2.34% (9)	2	5	0	2
Austria	High income	2.08% (8)	2	2	0	4
Belgium	High income	1.04% (4)	0	1	0	3
Brazil	Upper-middle income	0.26% (1)	0	1	0	0
Canada	High income	3.38% (13)	4	8	0	1
Chile	Upper-middle income	0.26% (1)	0	1	0	0
China	Upper-middle income	2.86% (11)	2	7	0	2
Denmark	High income	0.52% (2)	0	0	1	1
France	High income	6.75% (26)	3	12	1	10
Germany	High income	10.13% (39)	1	17	4	17
Greece	High income	0.52% (2)	0	2	0	0
Hong Kong	High income	0.26% (1)	0	0	0	1
Hungary	Upper-middle income	0.26% (1)	0	1	0	0
India	Lower-middle income	0.52% (2)	0	1	0	1
Iran	Upper-middle income	0.52% (2)	0	0	0	2
Israel	High income	0.78% (3)	1	1	1	0
Italy	High income	4.16% (16)	3	6	1	6
Japan	High income	5.97% (23)	0	17	0	6
Korea	High income	2.34% (9)	3	4	1	1
New Zealand	High income	0.26% (1)	0	0	0	1
Oman	Upper-middle income	0.26% (1)	0	1	0	0
Poland	Upper-middle income	0.26% (1)	0	0	0	1
Portugal	High income	0.26% (1)	0	1	0	0
Romania	Upper-middle income	0.52% (2)	0	0	2	0
Scotland	High income	0.26% (1)	0	1	0	0
Singapore	High income	0.52% (2)	1	1	0	0
Slovenia	High income	2.34% (9)	2	6	0	1
South Africa	Upper-middle income	0.26% (1)	0	0	0	1
Spain	High income	0.78% (3)	0	1	1	1
Sweden	High income	1.04% (4)	0	2	1	1
Switzerland	High income	3.12% (12)	1	6	2	3
Taiwan	High income	0.26% (1)	0	1	0	0
The Netherlands	Upper-middle income	2.6% (10)	1	7	0	2
Turkey	High income	0.52% (2)	0	1	0	1
United Arab Emirates	High income	0.26% (1)	0	1	0	0
United Kingdom	High income	5.45% (21)	4	7	1	9
United States of America	High income	36.1% (139)	18	54	14	53

Distribution of male and female editors stratified by World Bank income classification and editorial position. High-income countries dominate editorial participation, yet female inclusion remains inconsistent. Lower- and upper-middle-income countries contributed fewer editors overall, with markedly limited female representation

Differences in gender distribution across income groups were statistically significant (χ² = 9.22, df = 2, *p* = 0.0099), though no significant difference was observed between EIC and deputy roles (χ² = 1.43, *p* = 0.231).

### Predictive modeling of female representation

Multivariable logistic regression (predictors: Eigenfactor Score, JIF quartile, editorial role) identified Eigenfactor Score as a significant predictor of female representation (β = 19.96, *p* = 0.049). JIF quartile and editorial role were not independently associated.

**Fig. 3:**
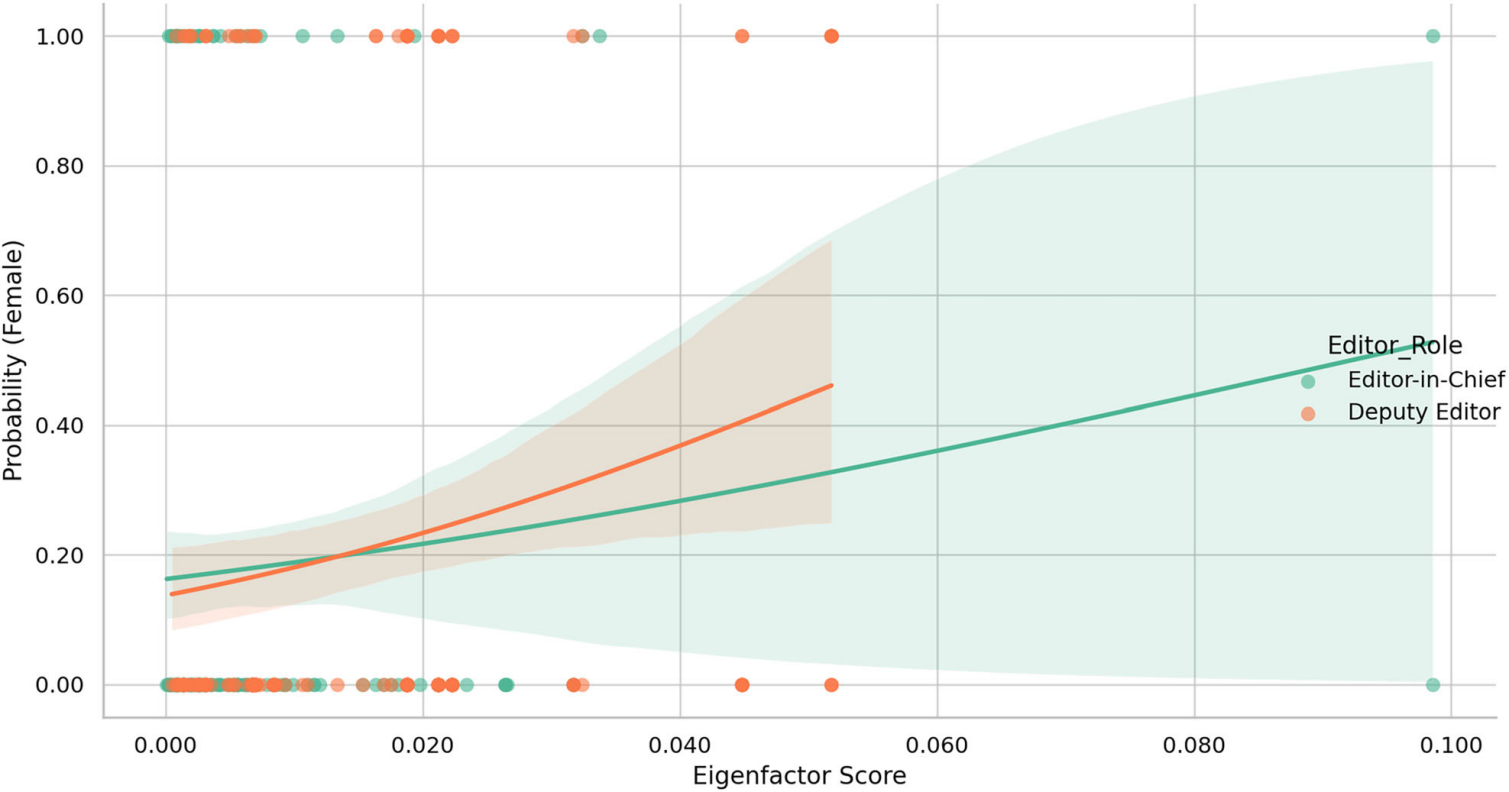
Predicted probability of female representation by Eigenfactor Score and editorial role. Logistic regression curves show the predicted probability of female inclusion as a function of Eigenfactor Score, stratified by editorial role. Female representation increases slightly with higher Eigenfactor Scores, more noticeably among deputy editors. Shaded areas represent 95% confidence intervals

The model demonstrated near-significant overall fit (likelihood ratio test, *p* = 0.064; pseudo R² = 0.027). Stratified logistic regression showed that the probability of female representation increased modestly with higher Eigenfactor Scores, with a slightly stronger trend among deputy editors (Fig. 3).

These results suggest that although disparities persist, bibliometric prestige may modestly enhance gender inclusivity, particularly within deputy editorial positions.

## Discussion

Gender equity within editorial boards remains a critical issue in academic publishing, particularly in radiology-related journals. Despite rising awareness, limited data exist on the proportion of positions held by women across all JCR-indexed radiology journals, making it difficult to monitor longitudinal trends through 2024. Previous studies have analyzed only subsets of journals—typically fewer than half of those in the “Radiology, Nuclear Medicine & Medical Imaging” category—leaving gaps in understanding how editorial gender representation relates to bibliometric performance and geographic diversity [7, 8]. Furthermore, the extent of inequity across high-income and low- and middle-income countries (LMICs) remains insufficiently described [7, 8].

Editorial board membership is a recognized marker of academic prestige, often influencing professional advancement and institutional recognition. Persistent gender disparities in these roles therefore signify more than numerical imbalance; they reflect entrenched systemic inequities [14]. Importantly, diversity is not only an ethical imperative but also a driver of academic excellence. Previous research demonstrates that inclusive teams produce higher-quality work, receive more citations, and attract greater funding [8, 15]. Global initiatives, such as The Lancet Group’s #LancetWomen campaign, have highlighted practical approaches to improve gender inclusion in editorial structures [16].

Our study provides added value by evaluating all radiology journals indexed in JCR 2024, thereby overcoming the limited coverage of earlier reports. For example, Alkhawtani et al [1] restricted analysis to journals with IF > 2.0, while Abdellatif et al [17] examined those affiliated with major radiology societies, reporting female representation of 8.8% among editors-in-chief and 19.1% among editorial board members. By incorporating all indexed journals, we present a broader, more representative picture of editorial gender disparity in radiology publishing. Additionally, by integrating both bibliometric indicators and World Bank economic classifications, we extend the analytical scope beyond prior investigations.

### Gender diversity in editorial roles

Our findings confirmed the underrepresentation of women, who comprised 21.4% of deputy editors and 18.4% of editors-in-chief. These results are consistent with prior evidence of male dominance in editorial leadership [1, 2]. While some regions reported encouraging improvements, structural barriers remain largely unresolved [6]. Equity benchmarks should consider the proportion of women in the radiology workforce within each country. For example, in the United States, only about 27% of radiologists are women [18], whereas in the United Kingdom, women account for 42% of diagnostic clinical radiology consultants [19]. Contextualized targets may therefore better capture true equity within academic leadership pipelines.

### Geographic disparities

Gender representation varied markedly across countries. Romania (*n* = 1, full representation), Israel (66.7%), and Denmark (50%) demonstrated relatively high female participation, while Japan, Brazil, and Belgium reported none. These differences may be related to diverse sociocultural norms, institutional policies, and advocacy structures. Regional frameworks modeled on initiatives such as #LancetWomen could help address these imbalances and accelerate progress [15].

### Impact of bibliometric and economic factors

Our multivariable model identified the Eigenfactor Score as a modest yet statistically significant predictor of female representation (β = 19.96, *p* = 0.049). This suggests that journals with higher scientific influence may be more likely to include women on editorial boards. In contrast, neither JIF quartile nor editorial role independently predicted female inclusion. This is consistent with our previous work, which emphasized the predictive and integrative value of alternative metrics such as the Article Influence Score and Cited Half-Life over traditional JIFs [11, 20, 21].

Figure 3 further illustrated that the Eigenfactor effect may differ by role, with a stronger association observed among deputy editors compared to editors-in-chief. This finding underscores persistent barriers to senior leadership—an expression of the “glass ceiling” long recognized in academic medicine [22, 23]. Multiple studies have confirmed these enduring gaps in radiologic leadership [6, 24–28].

Attempts to expand modeling with additional bibliometric variables (Article Influence Score, Immediacy Index, Total Citations) failed due to perfect separation, reflecting the stark imbalance in gender representation. This limitation itself highlights structural inequities rather than random variation [7, 8]. Notably, gender equity did not differ significantly across World Bank income categories, suggesting that national wealth alone does not drive inclusion. Instead, institutional culture, policy, and transparency may be more decisive.

Another structural factor that could influence gender representation is the financial remuneration of editorial roles. Some journals compensate editors, while others do not; however, this information is rarely disclosed publicly. Remuneration may affect who applies for or accepts editorial positions, and could disproportionately impact women, given existing workforce shortages and the rising demands of teleradiology with its reciprocal financial implications. Because of the lack of transparency on compensation policies, we were unable to evaluate this dimension systematically. Future studies should investigate the potential influence of remuneration on gender equity in editorial leadership, and journals should move toward greater transparency in disclosing compensation structures.

### World Bank country classification

While high-income countries accounted for the majority of editorial roles, the lack of statistically significant differences in gender distribution by income group implies that economic resources alone do not guarantee gender equity. Institutional policy and academic culture likely play a stronger role.

### Nominal logistic fit for gender

The Eigenfactor Score emerged as the only significant predictor of gender representation. Neither JIF quartile, income level, nor editorial role demonstrated statistical associations. These findings highlight the need to investigate non-bibliometric influences such as mentorship availability, leadership training, and gender-aware editorial selection policies.

### Limitations and future directions

This cross-sectional study provides a snapshot of editorial board composition rather than temporal trends. Reliance on publicly accessible listings introduces potential inaccuracies, particularly for rapidly changing boards. Another limitation is that our analysis necessarily adopted a binary definition of gender, excluding gender-diverse and non-binary identities, which remain underrepresented in editorial leadership. Future research should adopt inclusive classification frameworks and incorporate qualitative data on institutional policies and selection criteria to better characterize the drivers of inequity.

Another limitation relates to the absence of information regarding the financial remuneration of editorial roles. Some journals compensate editors, while others do not; however, this information is rarely disclosed publicly. Because of the lack of transparency on compensation policies, we were unable to evaluate this dimension systematically. Future studies should investigate the potential influence of remuneration on gender equity in editorial leadership, and journals should move toward greater transparency in disclosing compensation structures.

Looking ahead, progress will require greater transparency from journals themselves. Equity could be advanced if journals regularly reported the gender, geographic, and professional diversity of their editors, reviewers, and authors, and disclosed whether editorial positions are financially compensated. Lack of transparency may perpetuate inequities, including the “minority tax,” in which underrepresented groups contribute disproportionately without financial recognition. Considering the significant financial gains of many journals, adopting best practices for public reporting of diversity metrics and remuneration policies would foster accountability, guide authors in choosing journals aligned with their values, and accelerate systemic change in academic publishing.

## Conclusion

This study demonstrates that gender disparities remain entrenched within the editorial boards of radiology journals, with women consistently underrepresented in leadership positions. While high-income countries dominate editorial representation, economic resources alone do not guarantee equity. The Eigenfactor Score emerged as the only bibliometric factor modestly associated with female inclusion, underscoring the limited role of journal prestige in addressing systemic imbalance.

Bridging these gaps requires structural reforms beyond symbolic initiatives. Strategies such as dismantling the “glass ceiling”, developing mentorship pipelines, and fostering inclusive editorial cultures are essential.

Ultimately, gender equity in academic publishing is not simply an ethical obligation but a cornerstone of scientific excellence. Ensuring diverse participation in editorial governance will strengthen the quality, reach, and global relevance of radiology scholarship.

## Supplementary information

ELECTRONIC SUPPLEMENTARY MATERIAL

## Data Availability

The datasets generated and analyzed during the current study are available from the corresponding author (E.R.V.) upon reasonable request.

## Abbreviations

EIC: Editor-in-Chief
IF: Impact factor
JCR: Journal citation reports
JIF: Journal impact factor
JMP: JMP Statistical Software (SAS Institute)

## References

[1] Alkhawtani RHM, Kwee TC, Kwee RM (2021) Gender diversity among editorial boards of radiology-related journals. Clin Imaging 75:30–33 33493734 10.1016/j.clinimag.2021.01.007

[2] Campos LN, Naus A, Rangel AG et al (2022) Women representation on editorial boards in Latin America journals: promoting gender equity in academic surgery, anesthesia, and obstetrics. World J Surg. 47:845–853 10.1007/s00268-022-06872-8 36587176

[3] Nagano N, Watari T, Tamaki Y, Onigata K (2022) Japan’s academic barriers to gender equality as seen in a comparison of public and private medical schools: a cross-sectional study. Womens Health Rep (New Rochelle) 3:115–123 35136883 10.1089/whr.2021.0095 PMC8812492

[4] Hancı V, Yakar MN, Shermatov N et al (2024) The gender composition of the members of the editorial board of toxicology journals: assessment of gender equality. Basic Clin Pharmacol Toxicol 134:413–423 38030412 10.1111/bcpt.13968

[5] Dorrigan A, Zuccala E, Talley NJ (2022) Striving for gender equity at the *Medical Journal of Australia*. Med J Aust 217:138–139 35908263 10.5694/mja2.51642

[6] Kubik-Huch RA, Vilgrain V, Krestin GP et al (2020) Women in radiology: gender diversity is not a metric—it is a tool for excellence. Eur Radiol 30:1644–1652 31802213 10.1007/s00330-019-06493-1 PMC7033068

[7] Fox CW, Duffy MA, Fairbairn DJ, Meyer JA (2019) Gender diversity of editorial boards and gender differences in the peer review process at six journals of ecology and evolution. Ecol Evol 9:13636–13649

[8] D’Armiento J, Witte SS, Dutt K, Wall M, McAllister G, Columbia University Senate Commission on the Status of Women (2019) Achieving women’s equity in academic medicine: challenging the standards. Lancet 393:e15–e16 30739701 10.1016/S0140-6736(19)30234-X

[9] Krampl A (2019) Journal citation reports. J Med Libr Assoc 107:280–283

[10] Cuschieri S (2019) The STROBE guidelines. Saudi J Anaesth 13:S31–S34 30930717 10.4103/sja.SJA_543_18 PMC6398292

[11] Roldan-Valadez E, Salazar-Ruiz SY, Ibarra-Contreras R, Rios C (2019) Current concepts on bibliometrics: a brief review about impact factor, Eigenfactor score, CiteScore, SCImago Journal Rank, Source-Normalised Impact per Paper, H-index, and alternative metrics. Ir J Med Sci 188:939–951 30511320 10.1007/s11845-018-1936-5

[12] Heidari S, Fernandez DGE, Coates A et al (2024) WHO’s adoption of SAGER guidelines and GATHER: setting standards for better science with sex and gender in mind. Lancet 403:226–228 38134947 10.1016/S0140-6736(23)02807-6

[13] The World Bank Group (2024) World Bank country classifications by income level. The World Bank Group, Washington, DC. Available via https://blogs.worldbank.org/en/opendata/world-bank-country-classifications-by-income-level-for-2024-2025. Accessed 18 Aug 2024

[14] Gottlieb M, Krzyzaniak SM, Mannix A et al (2021) Sex distribution of editorial board members among emergency medicine journals. Ann Emerg Med 77:117–123 32376090 10.1016/j.annemergmed.2020.03.027

[15] Clark J, Horton R (2019) What is *The Lancet* doing about gender and diversity? Lancet 393:508–510 30739674 10.1016/S0140-6736(19)30289-2

[16] The Editors of The Lancet Group (2019) The *Lancet* Group’s commitments to gender equity and diversity. Lancet 394:452–453 31402014 10.1016/S0140-6736(19)31797-0

[17] Abdellatif W, Shao M, Jalal S et al (2019) Novel geographic thematic study of the largest radiology societies globally: how is gender structure biased within editorial boards? AJR Am J Roentgenol 213:2–7 30973771 10.2214/AJR.18.20965

[18] Henderson M (2023) Too few women in the field of radiology. Radiological Society of North America, Oak Brook, IL. Available via https://www.rsna.org/news/2023/april/increasing-number-of-female-radiologists. Accessed 9 Sep 2025

[19] The Royal College of Radiologists (2024) 2024 Workforce census. Clinical Radiology, London. Available via https://www.rcr.ac.uk/media/4imb5jge/_rcr-2024-clinical-radiology-workforce-census-report.pdf?utm_source=chatgpt.com. Accessed 9 Sep 2025

[20] Roldan-Valadez E, Orbe-Arteaga U, Rios C (2018) Eigenfactor score and alternative bibliometrics surpass the impact factor in a 2-years ahead annual-citation calculation: a linear mixed design model analysis of radiology, nuclear medicine and medical imaging journals. Radiol Med 123:524–534 29508240 10.1007/s11547-018-0870-y

[21] Villasenor-Almaraz M, Islas-Serrano J, Murata C, Roldan-Valadez E (2019) Impact factor correlations with Scimago Journal Rank, Source Normalized Impact per Paper, Eigenfactor Score, and the CiteScore in radiology, nuclear medicine & medical imaging journals. Radiol Med 124:495–504 30725395 10.1007/s11547-019-00996-z

[22] Zhuge Y, Kaufman J, Simeone DM, Chen H, Velazquez OC (2011) Is there still a glass ceiling for women in academic surgery? Ann Surg 253:637–643 21475000 10.1097/SLA.0b013e3182111120

[23] Babic A, Hansez I (2021) The glass ceiling for women managers: antecedents and consequences for work-family interface and well-being at work. Front Psychol 12:618250 33767646 10.3389/fpsyg.2021.618250 PMC7985459

[24] Hamidizadeh R, Jalal S, Pindiprolu B et al (2018) Influences for gender disparity in the radiology societies in North America. AJR Am J Roentgenol 211:831–838 30063373 10.2214/AJR.18.19741

[25] Qamar SR, Khurshid K, Jalal S et al (2020) Gender disparity among leaders of Canadian academic radiology departments. AJR Am J Roentgenol 214:3–9 31691610 10.2214/AJR.18.20992

[26] Surawicz CM (2016) Women in leadership: why so few and what to do about it. J Am Coll Radiol 13:1433–1437 28341310 10.1016/j.jacr.2016.08.026

[27] Battaglia F, Shah S, Jalal S et al (2019) Gender disparity in academic emergency radiology. Emerg Radiol 26:21–28 30194569 10.1007/s10140-018-1642-7

[28] Qamar SR, Khurshid K, Jalal S et al (2018) Academic musculoskeletal radiology: influences for gender disparity. Skelet Radiol 47:381–387 10.1007/s00256-017-2836-x 29260259

