# Supplementary material for: Global gender disparities in editorial leadership of radiology journals: a cross-sectional analysis of bibliometric and economic associations
Source: Insights Imaging. 2025 Oct 30;16:233. doi: 10.1186/s13244-025-02128-w (PMC12575894; doi:10.1186/s13244-025-02128-w)
Supplement: Supplementary file 1 — ELECTRONIC SUPPLEMENTARY MATERIAL [file 13244_2025_2128_MOESM1_ESM.pdf]

Global Gender Disparities in Editorial Leadership of Radiology Journals: A Cross-Sectional Analysis of Bibliometric and Economic Associations

ELECTRONIC SUPPLEMENTARY MATERIAL

Supplementary Table: Bibliometric Indicators for Selected Journals (JCR 2023)

Journal Title	2023 JIF	Immediacy Index	Eigenfactor	Article Influence Score	Cited Half-Life	Total Articles	Total Citations	JIF Without Self-Cites	Normalized Eigenfactor
ABDOMINAL RADIOLOGY	2.3	0.3	0.01148	0.643	3.9	275	6859	2.1	2.50807
ACADEMIC RADIOLOGY	3.8	1.3	0.00999	0.931	4.8	317	9023	3.1	2.18278
ACTA RADIOLOGICA	1.1	0.2	0.00348	0.343	8.7	144	4939	1.1	0.76092
AMERICAN JOURNAL OF NEURORADIOLOGY	3.1	0.7	0.01491	1.069	10.6	203	23271	2.9	3.25787
AMERICAN JOURNAL OF ROENTGENOLOGY	4.7	3.1	0.01603	1.157	12.7	97	32133	4.4	3.50239
ANNALS OF NUCLEAR MEDICINE	2.5	0.4	0.00276	0.591	6.3	54	2717	2.3	0.60482
APPLIED RADIATION AND ISOTOPES	1.6	0.4	0.00524	0.29	9.3	410	9886	1.3	1.1469
BIOMEDICAL OPTICS EXPRESS	2.9	0.9	0.01432	0.718	5.5	418	14436	2.4	3.12985
BMC MEDICAL IMAGING	2.9	0.4	0.00375	0.648	3.6	206	3062	2.9	0.81972
BRACHYTHERAPY	1.7	0.3	0.00212	0.423	6.0	91	2268	1.3	0.46436
BRITISH JOURNAL OF RADIOLOGY	1.8	1.0	0.01212	0.672	7.4	205	13451	1.8	2.64911
CANADIAN ASSOCIATION OF RADIOLOGISTS JOURNAL-JOURNAL DE L ASSOCIATION CANADIENNE DES RADIOLOGISTES	2.9	2.3	0.00201	0.741	4.3	68	1654	2.5	0.44018
CANCER BIOTHERAPY AND RADIOPHARMACEUTICALS	2.4	0.7	0.00204	0.481	5.0	25	2492	2.4	0.44607
CANCER IMAGING	3.5	0.7	0.00327	1.06	4.8	117	2885	3.4	0.71454
CANCER RADIOTHERAPIE	1.5	0.2	0.00119	0.239	4.6	78	1225	1.3	0.26069
CARDIOVASCULAR AND INTERVENTIONAL RADIOLOGY	2.8	0.8	0.00611	0.679	7.1	133	7200	2.5	1.33512
CIRCULATION-CARDIOVASCULAR IMAGING	6.5	1.7	0.0094	3.385	7.1	75	7458	6.3	2.05428
CLINICAL AND TRANSLATIONAL IMAGING	2.3	0.8	0.00082	0.471	4.6	8	737	2.1	0.17999
CLINICAL AND TRANSLATIONAL RADIATION ONCOLOGY	2.7	0.5	0.0039	0.961	3.5	130	1801	2.6	0.85298
CLINICAL IMAGING	1.8	0.2	0.00551	0.468	4.6	161	4061	1.7	1.20594
CLINICAL NEURORADIOLOGY	2.4	0.4	0.00314	0.786	3.9	72	1735	2.3	0.68768
CLINICAL NUCLEAR MEDICINE	9.6	3.4	0.00526	1.506	5.3	83	6483	6.3	1.15112
CLINICAL RADIOLOGY	2.1	0.6	0.00592	0.579	8.5	216	7774	2.0	1.29534
COMPUTERIZED MEDICAL IMAGING AND GRAPHICS	5.4	0.7	0.00461	1.312	4.9	109	4580	5.1	1.00785

CONCEPTS IN MAGNETIC RESONANCE PART A	0.4	0.1	8e-05	0.233	13.3	8	497	0.4	0.01818
CURRENT MEDICAL IMAGING	1.1	0.5	0.0012	0.2	3.4	146	1118	1.1	0.26286
CURRENT RADIOPHARMACEUTICALS	1.5	0.5	0.00074	0.601	5.6	26	632	1.3	0.16234
DENTOMAXILLOFACIAL RADIOLOGY	2.9	0.9	0.00238	0.694	8.8	61	4144	2.6	0.52208
DIAGNOSTIC AND INTERVENTIONAL IMAGING	4.9	4.7	0.00309	0.976	4.6	49	3240	3.6	0.67546
DIAGNOSTIC AND INTERVENTIONAL RADIOLOGY	1.4	0.3	0.0019	0.543	6.2	96	2003	1.4	0.41636
DOSE-RESPONSE	2.3	0.7	0.00223	0.454	4.7	49	2350	2.2	0.48744
EJNMMI PHYSICS	3.0	0.8	0.0026	1.008	3.5	72	1561	2.7	0.56813
EJNMMI RESEARCH	3.1	0.7	0.00415	0.867	4.7	99	2830	3.0	0.90783
EUROPEAN HEART JOURNAL-CARDIOVASCULAR IMAGING	6.7	1.2	0.01724	2.515	5.7	162	10763	5.8	3.76697
EUROPEAN JOURNAL OF NUCLEAR MEDICINE AND MOLECULAR IMAGING	8.6	1.5	0.02852	2.199	4.8	308	22997	7.8	6.23116
EUROPEAN JOURNAL OF RADIOLOGY	3.2	0.6	0.015	0.848	7.0	396	16891	3.0	3.27756
EUROPEAN RADIOLOGY	4.7	0.8	0.04442	1.299	4.5	808	35542	4.3	9.70466
HEALTH PHYSICS	1.0	0.5	0.00181	0.348	14.0	77	4185	0.9	0.39718
HELLENIC JOURNAL OF NUCLEAR MEDICINE	0.9	0.1	0.00039	0.21	6.2	47	467	0.9	0.08607
HUMAN BRAIN MAPPING	3.5	0.6	0.02873	1.772	8.0	316	25862	3.4	6.27684
IEEE TRANSACTIONS ON MEDICAL IMAGING	8.9	2.3	0.03777	2.966	5.6	307	33906	8.4	8.25181
INSIGHTS INTO IMAGING	4.1	0.9	0.0088	1.478	4.5	161	6165	4.0	1.92453
INTERNATIONAL JOURNAL OF CARDIOVASCULAR IMAGING	1.5	0.4	0.00638	0.577	5.2	209	4604	1.5	1.39558
INTERNATIONAL JOURNAL OF COMPUTER ASSISTED RADIOLOGY AND SURGERY	2.3	0.4	0.0068	0.742	4.8	232	5561	2.1	1.48683
INTERNATIONAL JOURNAL OF HYPERTHERMIA	3.0	0.4	0.00522	0.693	6.4	127	6082	2.6	1.141
INTERNATIONAL JOURNAL OF RADIATION BIOLOGY	2.1	0.5	0.00341	0.519	9.5	101	5487	1.9	0.74528
INTERNATIONAL JOURNAL OF RADIATION ONCOLOGY BIOLOGY PHYSICS	6.4	2.1	0.02828	1.88	11.3	301	41921	6.0	6.1788
INTERNATIONAL JOURNAL OF RADIATION RESEARCH	0.4	0.1	0.00035	0.087	4.3	165	410	0.3	0.07822
INTERVENTIONAL NEURORADIOLOGY	1.5	0.3	0.00307	0.489	5.2	179	2373	1.3	0.67208
INVESTIGATIVE RADIOLOGY	7.0	3.6	0.00664	1.672	6.3	75	7033	5.5	1.45102
IRANIAN JOURNAL OF RADIOLOGY	0.2	0.1	0.00019	0.066	7.6	26	372	0.2	0.04195
JACC-CARDIOVASCULAR IMAGING	12.8	6.5	0.03014	5.194	4.9	69	16404	12.1	6.58575
JAPANESE JOURNAL OF RADIOLOGY	2.9	0.6	0.0032	0.651	4.1	95	2519	2.6	0.7008
JOURNAL OF APPLIED CLINICAL MEDICAL PHYSICS	2.0	0.3	0.00604	0.524	4.7	317	5335	1.7	1.32049
JOURNAL OF BIOMEDICAL OPTICS	3.0	0.7	0.00593	0.685	9.7	162	13480	2.7	1.2974

JOURNAL OF CARDIOVASCULAR COMPUTED TOMOGRAPHY	5.5	1.5	0.00387	1.412	5.9	46	3046	4.9	0.84674
JOURNAL OF CARDIOVASCULAR MAGNETIC RESONANCE	4.2	nan	0.00744	2.023	7.3	17	6296	4.1	1.62725
JOURNAL OF CLINICAL ULTRASOUND	1.2	0.5	0.00142	0.244	10.8	113	2415	1.0	0.31211
JOURNAL OF COMPUTER ASSISTED TOMOGRAPHY	1.0	0.4	0.00197	0.331	15.4	123	4454	1.0	0.43142
JOURNAL OF CONTEMPORARY BRACHYTHERAPY	1.1	0.2	0.00091	0.286	5.0	46	858	0.9	0.20083
JOURNAL OF DIGITAL IMAGING	2.9	0.8	0.00553	0.956	4.9	146	5161	2.7	1.20846
JOURNAL OF INNOVATIVE OPTICAL HEALTH SCIENCES	2.3	0.8	0.00092	0.364	4.2	56	1079	1.5	0.20112
JOURNAL OF MAGNETIC RESONANCE IMAGING	3.3	1.0	0.01818	1.215	7.4	308	19673	2.9	3.97284
JOURNAL OF MEDICAL IMAGING AND RADIATION ONCOLOGY	2.2	0.2	0.00217	0.454	5.5	66	2010	2.1	0.47616
JOURNAL OF MEDICAL ULTRASONICS	1.9	0.3	0.00103	0.342	4.8	49	981	1.7	0.22642
JOURNAL OF NEUROIMAGING	2.3	0.4	0.00311	0.672	6.8	78	2928	2.2	0.68162
JOURNAL OF NEURORADIOLOGY	3.0	1.4	0.00178	0.82	5.1	56	1538	2.8	0.38932
JOURNAL OF NUCLEAR CARDIOLOGY	3.0	0.8	0.00627	0.778	4.6	90	4777	2.2	1.37068
JOURNAL OF NUCLEAR MEDICINE	9.1	2.6	0.02795	2.606	7.3	275	29679	8.3	6.10752
JOURNAL OF RADIATION RESEARCH	1.9	0.2	0.00229	0.468	8.2	109	3327	1.8	0.50083
JOURNAL OF RADIOLOGICAL PROTECTION	1.4	0.4	0.00165	0.349	6.7	77	1687	1.1	0.36061
JOURNAL OF THE AMERICAN COLLEGE OF RADIOLOGY	4.0	1.8	0.01115	1.527	5.3	135	7260	3.5	2.43613
JOURNAL OF THE BELGIAN SOCIETY OF RADIOLOGY	1.0	0.4	0.00048	0.382	4.9	20	347	1.0	0.10526
JOURNAL OF THORACIC IMAGING	2.0	0.7	0.00149	0.565	7.4	54	1685	1.8	0.32619
JOURNAL OF ULTRASOUND IN MEDICINE	2.1	0.4	0.00751	0.588	7.8	181	8702	1.9	1.64193
JOURNAL OF VASCULAR AND INTERVENTIONAL RADIOLOGY	2.6	0.7	0.00755	0.85	8.7	202	10224	2.1	1.64999
KOREAN JOURNAL OF RADIOLOGY	4.4	1.0	0.00594	1.058	5.2	79	5172	3.8	1.29925
MAGNETIC RESONANCE IMAGING	2.1	0.5	0.00591	0.677	10.6	152	7803	2.0	1.29213
MAGNETIC RESONANCE IMAGING CLINICS OF NORTH AMERICA	1.5	0.2	0.00082	0.425	7.9	54	1203	1.4	0.17923
MAGNETIC RESONANCE IN MEDICAL SCIENCES	2.5	0.7	0.00119	0.532	5.3	70	1170	2.2	0.25998
MAGNETIC RESONANCE IN MEDICINE	3.0	0.5	0.01969	0.923	11.0	362	29548	2.2	4.30192

MAGNETIC RESONANCE MATERIALS IN PHYSICS BIOLOGY AND MEDICINE	2.0	1.0	0.00203	0.669	6.7	59	1802	1.9	0.44466
MEDICAL DOSIMETRY	1.1	0.2	0.00076	0.283	7.1	51	952	1.0	0.16626
MEDICAL IMAGE ANALYSIS	10.7	1.7	0.02853	3.048	4.1	252	20810	10.1	6.23339
MEDICAL PHYSICS	3.2	0.5	0.02226	0.926	8.3	684	30140	2.7	4.86531
MEDICAL ULTRASONOGRAPHY	1.8	0.5	0.00111	0.391	5.6	37	1142	1.7	0.24308
MOLECULAR IMAGING	2.2	0.6	0.00055	0.654	10.2	6	975	2.2	0.12075
MOLECULAR IMAGING AND BIOLOGY	3.0	0.4	0.00373	0.706	5.3	78	3173	2.9	0.81601
NEUROIMAGE	4.7	0.9	0.09117	2.263	10.3	570	107771	4.3	19.91989
NEUROIMAGING CLINICS OF NORTH AMERICA	1.3	0.3	0.00116	0.608	9.0	45	1575	1.3	0.25538
NEURORADIOLOGY	2.4	0.4	0.00618	0.78	8.5	117	6559	2.3	1.35043
NMR IN BIOMEDICINE	2.7	0.5	0.00827	1.125	8.2	159	7848	2.4	1.80733
NUCLEAR MEDICINE AND BIOLOGY	3.6	0.7	0.00182	0.672	10.5	38	3368	3.4	0.3993
NUCLEAR MEDICINE COMMUNICATIONS	1.3	0.3	0.00193	0.29	7.8	124	2834	1.2	0.42382
NUKLEARMEDIZIN-NUCLEAR MEDICINE	1.0	0.3	0.00037	0.291	9.0	21	506	0.9	0.08287
PEDIATRIC RADIOLOGY	2.1	0.6	0.00638	0.654	8.4	180	7293	1.9	1.39529
PHOTOACOUSTICS	7.1	1.5	0.00395	1.538	2.8	129	2673	5.7	0.86344
PHYSICA MEDICA-EUROPEAN JOURNAL OF MEDICAL PHYSICS	3.3	0.5	0.00735	0.677	4.0	192	5690	2.7	1.60596
PHYSICAL AND ENGINEERING SCIENCES IN MEDICINE	2.4	0.4	0.00161	0.592	3.0	149	1152	2.2	0.35254
PHYSICS IN MEDICINE AND BIOLOGY	3.3	0.6	0.02006	0.891	9.2	565	28376	2.8	4.38323
PRACTICAL RADIATION ONCOLOGY	3.4	0.9	0.00455	0.965	5.0	105	2950	3.2	0.99433
QUANTITATIVE IMAGING IN MEDICINE AND SURGERY	2.9	0.3	0.00704	0.662	2.9	571	5279	2.2	1.54013
QUARTERLY JOURNAL OF NUCLEAR MEDICINE AND MOLECULAR IMAGING	1.3	0.8	0.00056	0.339	8.7	22	735	1.3	0.12391
RADIATION AND ENVIRONMENTAL BIOPHYSICS	1.5	0.5	0.0009	0.41	10.7	32	1521	1.5	0.19827
RADIATION ONCOLOGY	3.3	0.6	0.00943	0.947	5.8	180	8431	3.1	2.061
RADIATION PROTECTION DOSIMETRY	0.8	0.1	0.00217	0.186	11.9	353	6086	0.7	0.47442
RADIATION RESEARCH	2.5	0.8	0.00326	0.7	13.9	94	8176	2.2	0.71348
RADIOGRAPHICS	5.2	1.2	0.00916	1.86	10.0	117	15185	4.8	2.00186
RADIOLOGIA MEDICA	9.7	0.6	0.00555	0.928	3.3	142	5683	8.9	1.21378
RADIOLOGIC CLINICS OF NORTH AMERICA	2.1	0.4	0.00155	0.497	9.7	76	2465	2.1	0.34036
RADIOLOGIE	0.7	0.2	0.00056	0.125	5.2	83	648	0.6	0.1231
RADIOLOGY	12.1	7.0	0.04751	3.816	9.0	263	59748	11.1	10.3816
RADIOLOGY AND ONCOLOGY	2.1	0.3	0.00145	0.588	4.9	51	1502	2.0	0.31821
RADIOPROTECTION	1.4	0.8	0.00029	0.175	6.9	38	538	0.4	0.06464
RADIOTHERAPY AND ONCOLOGY	4.9	1.1	0.02285	1.515	6.6	330	21942	4.4	4.99335
REVISTA ESPANOLA DE MEDICINA NUCLEAR E IMAGEN MOLECULAR	1.6	0.3	0.00041	0.246	6.2	42	528	1.4	0.0914
ROFO-FORTSCHRITTE AUF DEM GEBIET DER RONTGENSTRAHLEN UND	1.3	0.1	0.00133	0.463	6.9	128	1792	1.2	0.29217

DER BILDGEBENDEN VERFAHREN									
SEMINARS IN INTERVENTIONAL RADIOLOGY	1.0	0.1	0.00129	0.501	9.2	68	1863	0.9	0.28397
SEMINARS IN MUSCULOSKELETAL RADIOLOGY	0.9	0.3	0.00108	0.431	7.9	62	1280	0.9	0.23596
SEMINARS IN NUCLEAR MEDICINE	4.6	2.6	0.00248	1.102	6.8	0	2980	4.4	0.54319
SEMINARS IN RADIATION ONCOLOGY	2.6	0.5	0.00193	1.119	9.0	44	2645	2.6	0.42166
SEMINARS IN ROENTGENOLOGY	0.8	0.4	0.0002	0.157	11.0	26	370	0.8	0.04406
SEMINARS IN ULTRASOUND CT AND MRI	1.5	0.3	0.00094	0.479	8.9	44	1439	1.5	0.20562
SKELETAL RADIOLOGY	1.9	0.4	0.00548	0.545	9.3	171	7247	1.7	1.19859
STRAHLENTHERAPIE UND ONKOLOGIE	2.7	0.6	0.00285	0.614	6.7	93	3545	2.1	0.62368
SURGICAL AND RADIOLOGIC ANATOMY	1.2	0.4	0.00282	0.328	9.6	176	4622	1.0	0.6168
TOMOGRAPHY	2.2	0.6	0.00177	0.497	2.2	131	1293	2.0	0.38683
ULTRASCHALL IN DER MEDIZIN	3.1	0.5	0.00219	0.991	6.9	59	2760	2.8	0.47925
ULTRASONIC IMAGING	2.5	0.2	0.00042	0.505	20.8	24	1000	2.4	0.09326
ULTRASONICS	3.8	0.9	0.00607	0.703	7.7	246	10137	3.0	1.32729
ULTRASONOGRAPHY	2.4	0.4	0.00158	0.701	4.6	33	1146	2.2	0.34521
ULTRASOUND IN MEDICINE AND BIOLOGY	2.4	0.5	0.00862	0.705	8.5	218	12278	2.2	1.88443
ULTRASOUND IN OBSTETRICS & GYNECOLOGY	6.1	2.1	0.01512	2.039	8.1	149	16824	5.3	3.30492
ULTRASOUND QUARTERLY	0.7	0.5	0.00062	0.263	6.8	38	768	0.7	0.13635
ZEITSCHRIFT FUR MEDIZINISCHE PHYSIK	2.4	1.8	0.00118	0.85	4.6	31	992	2.2	0.25865
